# PAK1 regulates cortical development via promoting neuronal migration and progenitor cell proliferation

**DOI:** 10.1186/s13041-015-0124-z

**Published:** 2015-06-05

**Authors:** Xingxiu Pan, Xinxia Chang, Celeste Leung, Zikai Zhou, Feng Cao, Wei Xie, Zhengping Jia

**Affiliations:** The Key Laboratory of Developmental Genes and Human Disease, Ministry of Education, Institute of Life Sciences, Jiangsu Co-innovation Center of Neuroregeneration, Southeast University, 2 Sipailou Road, 210096 Nanjing, China; Neurosciences & Mental Health, The Hospital for Sick Children, 555 University Ave., M5G 1X8 Toronto, Ontario Canada; Department of Physiology, Faculty of Medicine, University of Toronto, Toronto, Canada

**Keywords:** PAK1, Cerebral cortex, Neuronal migration, Cell proliferation, Cell cycle

## Abstract

**Background:**

p21-activated kinase 1 (PAK1) is a serine/threonine kinase known to be activated by the Rho family small GTPases and to play a key role in cytoskeletal reorganization, spine morphology and synaptic plasticity. PAK1 is also implicated in a number of neurodevelopmental and neurodegenerative diseases, including autism, intellectual disability and Alzheimer’s disease. However, the role of PAK1 in early brain development remains unknown.

**Results:**

In this study, we employed genetic manipulations to investigate the role of PAK1 in the cerebral cortical development in mice. We showed that compared to the wild type littermates, PAK1 knockout mice have a reduction in the number of pyramidal neurons in several layers of the cerebral cortex, which is associated with a smaller pool of neural progenitor cells and impaired neuronal migration.

**Conclusion:**

These results suggest that PAK1 regulates cortical development by promoting the proliferation of neural progenitor cells and facilitating the migration of these neurons to specific regions of the cortex.

## Background

In the mammalian CNS, the cerebral cortex is a key region that dictates brain volume and overall organization. Cortical defects are associated with many neurological and mental disorders, including epilepsy, schizophrenia, autism and intellectual disability [1–4]. The development of the cerebral cortex requires a series of highly complex and regulated events that include the amplification of radial glia cells (RGCs), generation of neurons at the subventricular zone, their migration to specified cortical regions, and finally their differentiation and maturation into functioning cells [3–5]. Ample studies have shown that neurogenesis (i.e. generation of neurons) is the predominant determinant for cortical growth and organization. Thus, a number of proteins (e.g. LIS and Nde) that are important for mitotic cell division and survival have been shown to be critical for normal cortical formation and organization [5]. Similarly, proteins required for neuronal migration, particularly those involved in the Reelin and CDK5 protein kinase signal transduction pathways, are equally important for normal cortical development [4, 5]. Exactly how these molecules regulate neuronal migration and what cellular processes are required remain unknown. Given the importance of the actin cytoskeleton in the regulation of cell morphology and motility, it has long been suggested that Reelin/CDK5 may control neuronal migration through actin reorganization [4, 5], but the molecules that are responsible for linking Reelin/CDK5 to actin remain elusive.

p21 activated kinases (PAKs) are a family of serine/threonine proteins kinases known to be involved in multiple cellular processes, including actin reorganization, cell proliferation, cell morphology, motility and structural remodeling [6–9]. In neurons, we and others have shown that PAKs are important for neurite outgrowth, spine morphology, synaptic plasticity and learning and memory [9–22]. However, most of these studies have been focused on cultured cell lines or adult animals, and therefore whether or not PAKs play a role in the developing brain remains unknown. However, a number of studies exist that support this possibility. First, PAKs are highly expressed in the developing brain, including neurons [7, 21, 23]. Second, the kinase activity of PAKs can be regulated by CDK5 and Rreelin [6, 7, 24], both of which are critically involved in cortical development. Third, studies in lower invertebrates indicate the involvement of PAKs in neuronal development [25–28]. Finally, PAK gene mutations are closely linked to human cortical malformations such as microcephaly [29, 30]. Indeed, a recent study using shRNA to knockdown PAK1 suggests that PAK1 is required for neuronal migration, but whether it is also involved in progenitor cell proliferation remains unknown [13]. In this study, we used a genetic approach to investigate the role of PAK1, the predominant member of the PAK family expressed in the brain. We demonstrate that PAK1 plays a key role in both progenitor cell proliferation and neuronal migration during cerebral cortical development.

## Results

### PAK1 is expressed in both mitotic and post-mitotic neurons in the developing cortex

**Fig. 1:**
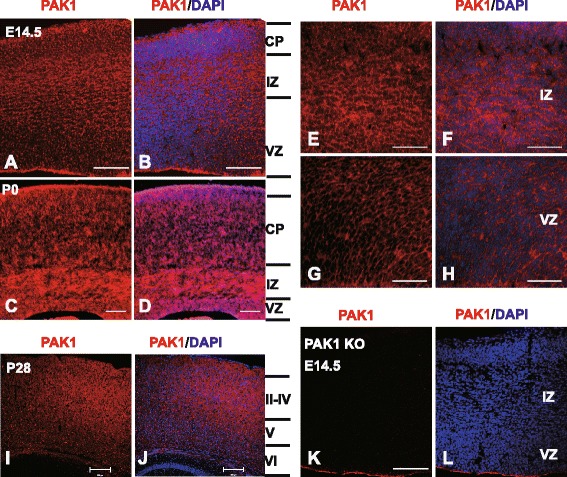
PAK1 expression in the developing mouse neocortex. Coronal brain sections of E14.5, P0 and P28 were stained with anti-PAK1 (red) and DAPI (blue). **a**, **b** PAK1/DAPI staining of WT E14.5 dorsal telencephalon. CP, cortical plate; VZ, ventricular zone; IZ, intermediate zone. **c**, **d** PAK1/DAPI staining of WT P0 neocortical sections. **e**-**h** Magnified regions of IZ and VZ areas in **a** and **b**. **i**, **j** PAK1/DAPI staining of WT P28 coronal sections. **k**, **l** PAK1/DAPI staining of PAK KO E14.5 telencephalon showing the absence of PAK1 immunostaining signals. Scale bars: 100 μm (**a**-**d**, **k** and **l**), 200 μm for (**i** and **j**), 50 μm (**e-h**)

In order to investigate the role of PAK1 in cortical development, we first analyzed the expression of PAK1 in developing brain using antibodies specific to PAK1 through immunohistochemistry. Three developmental stages, embryonic day (E) 14.5, postnatal day (P) 0 and P28 were examined. During early development (E14.5) of the telencephalon (Fig. 1A, B and E-H), PAK1 was detected in all the areas, of which the intermediate zone (IZ) has the highest expression level, followed by the cortical plate (CP) and the ventricular zone (VZ)/subventricular zone (SVZ). The high level of expression of PAK1 in post-mitotic neurons in IZ and CP are consistent with its demonstrated roles in regulating the morphology and migratory properties of the neurons [13]. In addition, strong signals were detected in the VZ/SVZ layers where the neuronal precursor cells reside, suggesting that PAK1 may also play an important role in the proliferation and differentiation of these cells. Similarly, at P0 (Fig. 1C, D), PAK1 was found to be highly expressed in VZ and IZ but a lower level was detected in the CP area. In the cerebral cortex of the mature mouse (P28, Fig. 1I, J), PAK1 expression was detectable in all cortical layers, reflecting its roles in synaptic transmission and plasticity. The PAK1 antibodies were highly specific because no immunostaining signals were detected in PAK1 KO mice (Fig. 1K, L). The widespread expression of PAK1 in all cortical layers in early embryonic stages suggests that PAK1 may play multiple roles during cortical development.

### Loss of PAK1 reduces the number of pyramidal neurons, particularly in the upper layers of the cortex

**Fig. 2:**
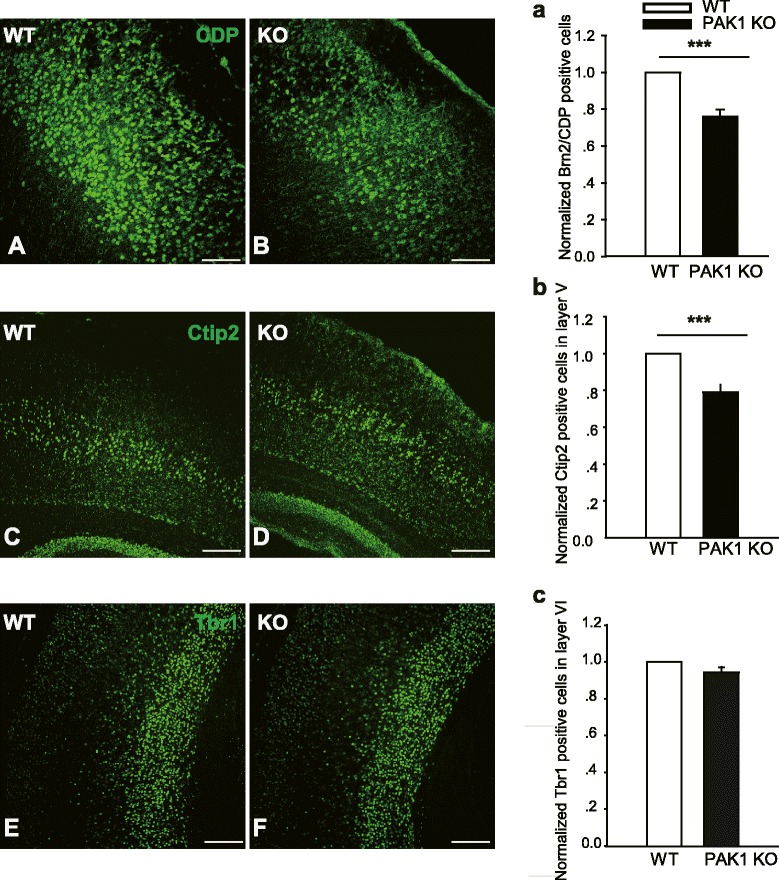
Reduced pyramidal neurons in PAK1 KO P7 cortex. **a**, **b** Coronal brain sections from WT (**a**, **c**, **e**) and PAK1 KO (**b**, **d**, **f**) P7 mice were stained for the late-born neuronal marker CDP (**a**, **b**) and for early-born neuronal markers, Ctip2 (**c**, **d**) and Tbr1 (**e**, **f**), respectively. **a**, **b**, **c** Summary graphs of CDP, Ctip2 and Tbr1 positive neurons of WT and PAK1 KO littermates showing a significant reduction in the number of neurons in layer II-V, but not in the Layer VI of the KO mice. ***p < 0.001. **p < 0.01. Scale bars: 100 μm

Having established the expression pattern of PAK1 in the developing cerebral cortex, we next investigated whether the absence of PAK1 affects the cortical organization by comparing PAK1 KO mice and their wild type (WT) littermates. Since neuronal migration ensures that the correct number and types of neurons are deposited in the appropriate cortical layer, we used layer specific markers (e.g. transcription factors specifically expressed in each layer) to examine the cortical distribution at P7. At this time point, neurons are known to have already migrated to their final destinations within the cortex. we found that the overall laminar organization of the cortex is normal in PAK1 KO mice. However, a detailed analysis of each cortical layer revealed a significant reduction (about 24 %) in the number of neurons in the superficial layer (identified by CDP and Brn2 staining) in PAK1 KO mice (0.759 ± 0.039; n = 3 pairs of littermates, p < 0.001) compared to their WT littermates (Fig. 2A, B and a). A significant reduction (about 21 %) in the number of neurons in layer V (by Ctip2 staining) was also found in PAK1 KO mice (Fig. 2C, D and b; KO: 0.790 ± .041; n = 3 pairs of littermates, p < 0.001). Interestingly, the number of layer VI neurons (by Tbr1 staining) remained the same between the WT and KO mice (Fig. 2E, F and c; KO: 0.942 ± .029; n = 3 pairs of littermates, p > 0.05). Similar results were obtained for the P0 brains where the Brn2 and CDP positive neurons were clearly reduced in PAK1 KO mice (data not shown). These results suggest that PAK1 is critical for neuronal proliferation, differentiation and/or migration during early cortical development.

### PAK1 is required for normal neuronal migration to the upper cortical layers

**Fig. 3:**
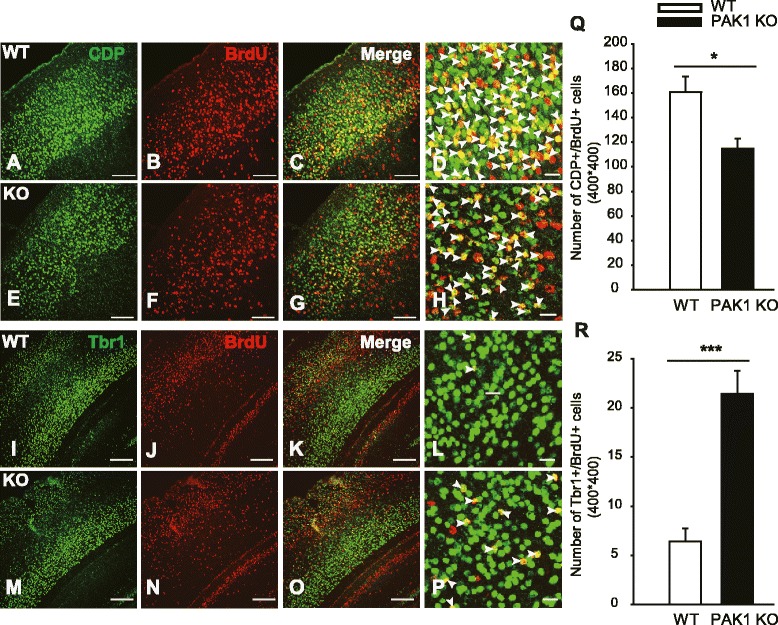
Reduced late-born pyramidal neurons in PAK1 KO P7 cortex. **a**–**h** Coronal brain sections from WT (**a**–**d**) and PAK1 KO (**e**–**h**) P7 mice were stained for the late-born neuronal marker CDP (green) and BrdU (red) injected at E14.5, showing reduced colocalization of CDP and BrdU (yellow) in PAK1 KO mice. **i-p** Coronal sections of the cortex of WT (**i**-**l**) and PAK1 KO (**m**-**p)** P7 mice stained for early-born neuronal markers, Tbr1 (green) and BrdU (red) injected at E14.5, showing increased colocalization of Tbr1 and BrdU. **d**, **h** and (**l**, **p**) are the magnified regions from the upper and deep layers marked by CDP and Tbr1, respectively. Arrowheads in (**d**, **h**) indicate CDP+/BrdU+ cells, in (**l**, **p**) indicate Tbr1+/BrdU+ cells. **q** Summary graph of CDP+/BrdU+ cells in WT and PAK1 KO mice. **r** Summary graph of Tbr1+/BrdU+ cells in WT and PAK1 KO mice. *p < 0.05, ***p < 0.001. Scale bars: 100 μm (**a**-**c**, **e**-**g**, **i**-**k**, **m**-**o**), 20 μm (**d**, **h**, **l**, **p**)

**Fig. 4:**
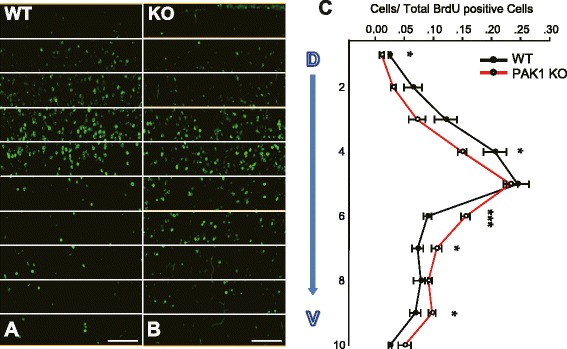
Impaired neuronal migration in PAK1 KO mice. **a**, **b** Pregnant mice were injected with BrdU at E14.5 and the brains of the WT (**a**) and PAK1 KO littermates (**b**) were dissected at P7 and stained with anti-BrdU antibodies. **c** Summary graph showing a significant shift of migrating cells to the deep layers of the cortex in PAK1 KO mice. *p < 0.05, ***p < 0.001

Cortical pyramidal neurons migrate in an “inside- out” manner, with the early born neurons targeting to the deep layers and the late born neurons migrating mainly to the upper more superficial layers [4, 5]. Thus, the reduced neuronal number in the upper layers in PAK1 KO mice suggests a deficit in neurons born around E14.5, in their proliferation/differentiation/survival and/or migration. To investigate whether the reduced CDP+ cell numbers were caused by a migration deficit, we utilized the BrdU injection assay. The pregnant mice were injected with BrdU at E14.5, and the number of BrdU+/CDP+ and BrdU+/Tbr1+ (positive for both BrdU and CDP or Tbr1) neurons of the somatosensory cortex were analyzed at P7. As shown in Fig. 3A-H and Q, the number of BrdU+/CDP+ neurons was clearly decreased in PAK1 KO compared to WT mice (WT: 155.800 ± 11.015; KO: 114.750 ± 8.350; n = 2 pairs of littermates, p < 0.03). In contrast, the number of BrdU+/Tbr1+ neurons was increased in the KO mice (Fig. 3I-P and R; WT: 6.429 ± 1.288; KO: 21.400 ± 2.372; n = 2 pairs of littermates, p < 0.001). These results suggest a delayed migration of E14.5 BrdU labeled neurons. To quantify the migration path, we divided the neocortex equally into 10 bins and analyzed the percentage of BrdU positive cells in each bin to obtain a distribution pattern of the neurons (Fig. 4). In WT mice, approximately 66 ± 3.1 % BrdU+ cells migrated to the upper layers, whereas only 50 ± 1.1 % BrdU+ cells migrated to the same area in the KO mice. These results together indicate that PAK1 is required for promoting neuronal migration of the late born neurons to their destinations.

### PAK1 acts as a positive regulator of apical progenitor cell proliferation

**Fig. 5:**
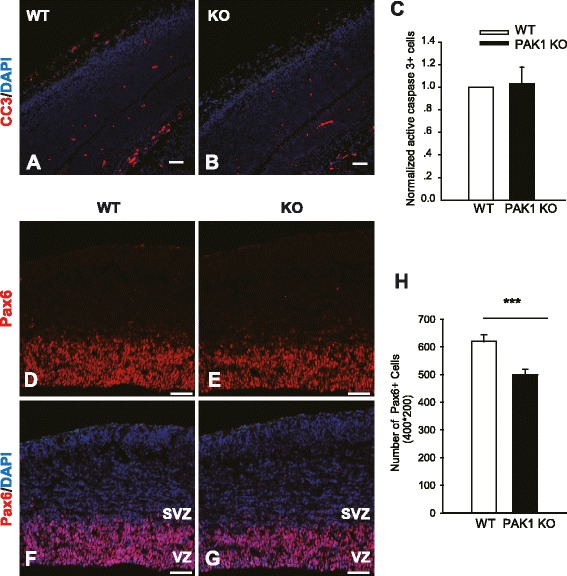
Reduced progenitor cells in PAK1 KO mice. Coronal brain sections of WT and PAK1 KO E14.5 embryos were stained for the active form of caspase 3 (CC3) or Pax6. **a**, **b** CC3 (red) and DAPI (blue) staining of WT (**a**) and PAK1 KO mice (**b**). **c** Summary graph of CC3 positive cells showing no differences between WT and PAK1 KO mice. **d**-**g** Pax6 (red) and DAPI (blue) staining of WT (**d**, **f**) and PAK1 KO mice (**e**, **g**). **h** Summary graph showing significantly decreased numbers of Pax6 positive cells PAK1 KO mice compared to their WT littermates. ***p < 0.001. Scale bars: 100 μm

**Fig. 6:**
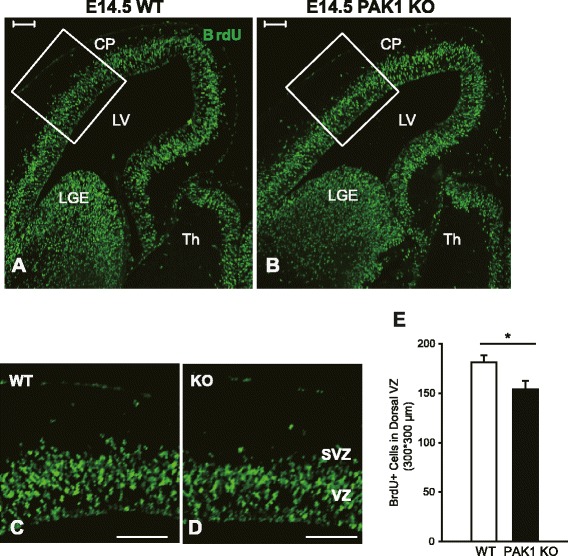
Decreased cell proliferation in the dorsal telencephalon of PAK1 KO mice. **a**, **b** Pregnant mice were labeled with BrdU at E14.5 for 2 h and the embryos were dissected and stained for BrdU. **c**, **d** Magnified views of the boxed regions in **a** and **b**. **e** Summary graph showing a significant reduction of BrdU positive cells in the dorsal VZ in PAK1 KO embryos compared with the WT littermates. ***p < 0.001. Scale bars: 100 μm. CP, cortical plate; LV, lateral ventricles; LGE, lateral ganglionic eminences; TH, thalamus

One possibility that may also result in reduced pyramidal neurons in PAK1 KO mice is that the KO mice have enhanced cell death. Therefore, we examined the degree of apoptotic cell death by performing immunostaining using the apoptotic cell death marker, the active form of caspase 3, but found no differences between PAK1 KO and WT mice (Fig. 5A-C; KO: 1.032 ± 0.147; n = 3 pairs of littermates, p > 0.05), suggesting that the cell death is not likely a significant factor contributing to the reduced neuronal number in the KO mice. Another possibility is that PAK1 KO mice may have less neurons generated in the first place due to impaired cell proliferation, given that PAK1 is expressed in the proliferating VZ/ SVZ zones (Fig. 1A, B and G, H). To address this possibility, we analyzed the apical neuronal progenitor cells in the dorsal VZ of the E14.5 brain using the RGC marker Pax6. An approximately 19 % decrease in the number of the apical progenitors was observed in PAK1 KO embryos compared to their WT controls (Fig. 5D-H; WT: 619.500 ± 24.592; KO: 499.750 ± 19.104; n = 4 pairs of littermates, p < 0.001). To determine whether the reduced progenitor cells were related to impaired cell division, we again injected the pregnant mice at E14.5 with a higher concentration of BrdU (200 mg/kg) for a brief period of time (2 h). The proliferating progenitor cells during the 2 h incubation period were labeled with BrdU. The embryos were then dissected and the BrdU+ cells were analyzed. We found a 15 % decrease in BrdU+ cells in the dorsal VZ in PAK1 KO compared to their WT littermate embryos (Fig. 6; WT: 181.182 ± 7.298; KO: 154.000 ± 8.452; n = 4 pairs of littermates, p < 0.02). Taken together, these results indicate that PAK1 is also an important positive regulator for cell proliferation of the apical progenitor cells.

### PAK1 regulates cell cycle progression

**Fig. 7:**
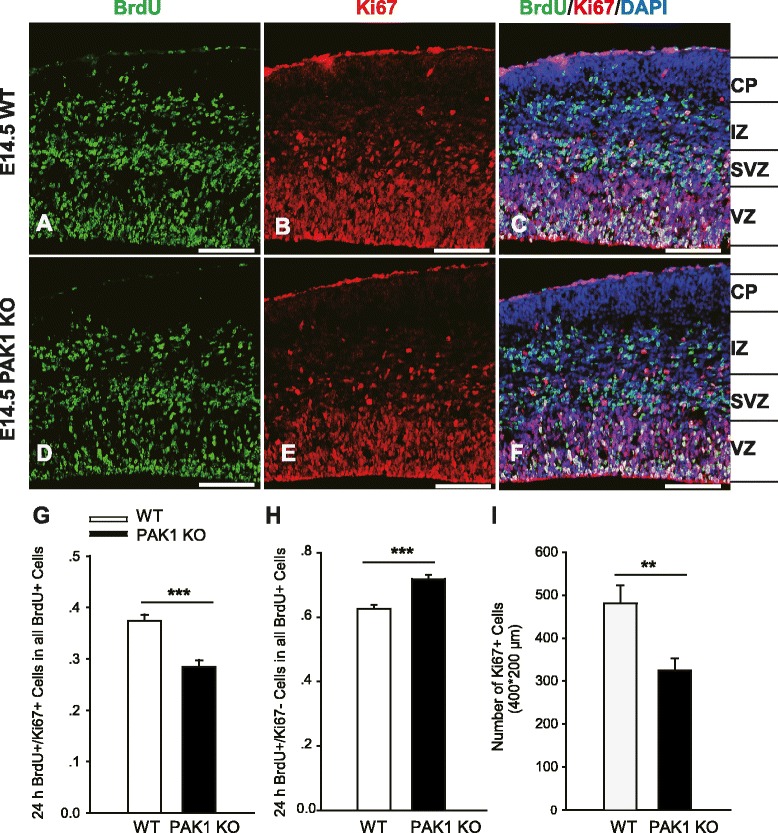
Altered cell cycle state in the telencephalon of PAK1 KO embryos. **a-f** Pregnant mice were injected with BrdU at E13.5 for 24 h and the embryos of the WT (**a**-**c**) and PAK1 KO (**d**-**f**) were then dissected at E14.5 and processed for immunostaining for BrdU (green), Ki67 (red) and DAPI (blue). **g**, **h** Summary graphs of the percentages of BrdU+/Ki67+ (**g**) and BrdU+/Ki67- (**h**) cells relative to the total BrdU+ cells showing a significantly decreased number of cells staying in the active phases of the cell cycle, as indicated by reduced BrdU+/Ki67+ cells and increased BrdU+/Ki67- cells, in PAK1 KO mice compared to the WT littermates. (**i**) Summary graph showing that the number of total Ki67 positive cells was also decreased in the dorsal telencephalon in the PAK1 KO mice compared to the WT littermates. **p < 0.01, ***p < 0.001. Scale bars: 100 μm. CP, cortical plate; VZ, ventricular zone; SVZ, subventricular zone; IZ, intermediate zone

To further investigate whether an altered cell cycle state (active or inactive) may contribute to the reduced pool of proliferating progenitor cells in PAK1 KO mice, we examined the expression of Ki67, a marker for all the active phases of the cell cycle. We injected the pregnant mice at E13.5 with a low concentration of BrdU (50 mg/kg) and 24 h later, the embryos were dissected and stained with antibodies against BrdU and Ki67 (Fig. 7). We found that the number of BrdU+/Ki67+ cells was significantly reduced by 23 % in PAK1 KO embryos compared to their WT littermate controls (Fig. 7G; WT: 0.374 ± 0.012; KO: 0.284 ± 0.013; n = 4 pairs of littermates, p < 0.001). In addition, the total number of Ki67+ cells was also reduced by 32 % in the KO embryos (Fig. 7I; WT: 480.750 ± 42.343; KO: 324.250 ± 28.142; n = 4 pairs of littermates, p < 0.01). These results indicate that a fewer number of cells in PAK1 KO mice were maintained in the active phases of the cell cycle. Consistent with this finding, the number of cells exiting the cell cycle (i.e. BrdU+/Ki67- cells) was increased by 13 % (Fig. 7H; WT: 0.626 ± 0.013; KO: 0.718 ± 0.014; n = 4 pairs of littermates, p < 0.001). Therefore, PAK1 is important for maintaining the apical progenitor cells in a proliferative state.

## Discussion

Abundant evidence indicates PAK1 is a critically involved in postnatal neuronal development, synaptic plasticity and cognitive processes [6–9], but little is known about its role in early brain development. In this study, we utilized genetically altered mice lacking PAK1 to specifically probe its role in the development of the cerebral cortex. We show that PAK1 plays a key role in ensuring proper number and type of pyramidal neurons in the cortex likely through maintaining an active pool of proliferative progenitor cells and facilitating neuronal migration. This conclusion is based on following lines of evidence.

First, we show that PAK1 is expressed in all cortical layers from E14.5 to P0. This is an important finding because although the expression of PAK1 in mature neurons and adult brain are well documented, its expression in mitotic cells during early brain development is unclear. Using PAK1 KO mice as a control for the specificity of the PAK1 antibodies used in this study, we conclusively demonstrate that PAK1 is expressed across all cortical layers, including VZ/SVZ zones where the proliferating progenitor cells reside. The widespread neuronal expression of PAK1 suggests that PAK1 may be involved in multiple stages of cortical neuronal development from the birth of the neurons to their migration to the final destination within the cortex.

Second, we demonstrate that PAK1 is required for normal neuronal migration to the upper layers of the cortex. In PAK1 KO mice, the distribution of BrdU positive neurons labeled at E14.5 is significantly shifted to the deep layers, indicating that migration of these neurons is impaired in the KO mice. These results are consistent with an early study in rats where downregulation of PAK1 by using viral expression of a shRNA against PAK1 delays neuronal migration and decreases neuronal number in layers II-IV [13]. This confirms that PAK1 is a key regulator for proper neuronal migration in the mammalian cortex. The mechanisms by which PAK1 regulates neuronal migration are unclear although the shRNA study suggests that PAK1 may be important for regulating the morphology of the migratory cells [8, 13]. Therefore, it would be important to see whether the morphology of the RGC or migratory cells are altered in PAK1 KO mice. Given the importance of PAK1 in cytoskeletal regulation, it is not surprising that cell morphology mediated by PAK1 may play a key role in the migration process. In addition, how PAK1 interacts with other molecules known to be involved in neuronal migration remains unknown. Of particular interest are CDC42, N-cadherin and Cdk5, all of which are shown to regulate PAK1 activity biochemically [6, 7, 24]. For example, PAK1 can be directly phosphorylated by Cdk5, a key player in morphological dynamics of migratory neurons and cortical development [24]. It is possible that PAK1 may act as a common effector of these molecules by regulating cytoskeletal reorganization and neuronal morphology during the migration and differentiation of cortical neurons.

Finally, we have revealed that PAK1 is also involved in the proliferation of early progenitor cells. In PAK1 KO mice, there is a smaller pool of Pax6 positive cells at E14.5 accompanied by no changes in the degree of apoptotic cell death. In addition, the number of cells labeled with brief BrdU injections at E13.5 is also reduced in the KO mice. These results are consistent with the notion that PAK1 is important for the proliferation of the progenitor cells during neurogenesis. This is an important finding because most of the early studies have been focused on post-mitotic neurons in the adult brain [8, 9]. However, it is known that in fibroblasts cell cycle arrest can be induced by manipulating PAK1 and that PAK1 can regulate cell cycle proteins such as cyclin D1 and D2 [31]. Therefore, the present study has extended previous in vitro results to mouse models, establishing PAK1 as an important in vivo regulator of neuronal proliferation

## Conclusions

In summary, we show here that PAK1 KO mice are significantly impaired in progenitor cell proliferation and neuronal migration. These results indicate that PAK1 is critical for cortical development by promoting proper cell division and neuronal migration.

## Methods

### Mice

The generation and initial characterization of PAK1 KO mice were described previously [10, 18]. To minimize the effect of genetic background, all the pups and embryos used in this study were PAK1 KO and their WT littermates from heterozygous PAK1+/− breeders. All the procedures were in accordance with animal regulations in the Hospital for Sick Children in Canada and Southeast University in China.

### Tissue processing and Immunohistochemistry

Standard procedures for brain processing and immunohistochemistry were described previously [32]. Briefly, postnatal day or E14.5 pregnant mice were anaesthetized with 10 % chloral hydrate or hypothermia and then subjected to cardiac perfusion with 0.1 M phosphate-buffered saline (PBS), followed by 4 % paraformaldehyde (PFA) dissolved in 0.1 M PBS. Brains were removed and post-fixed in 4 % PFA for 8–12 h for prenatal day mice and 12–24 h for postnatal day mice, and then replaced with 30 % sucrose dissolved in 0.1 M PBS at 4 °C until the brains were saturated. The brains were then embedded in Tissue-Tek® O.C.T. Compound and frozen by liquid nitrogen before being cut into coronal sections of 8 μm for prenatal day brains and 16 μm for postnatal day brains using a Leica CM 1950 cryostat.

For immunohistochemistry, brain sections were washed in 0.1 M PBS for 5 min, permeabilized with 0.2 % Triton X-100 dissolved in 0.1 M PBS (PBT), and then blocked with 10 % fetal bovine serum and 0.2 % Triton X-100 dissolved in 0.1 M PBS for 2 h before being incubated with primary antibodies overnight at 4 °C. Subsequently, sections were washed in 0.2 % PBT and incubated in appropriate secondary antibodies for 2 h at 37 °C. The primary antibodies used in this study included: rabbit anti-Ki67 (Abcam, 1:500), rabbit anti-Tbr1 (Proteintech Group, 1:500), rabbit anti-Ctip2 (Abcam, 1:1000), rabbit anti-Pax6 (Proteintech Group, 1:1000), rabbit anti-Pak1 (Abcam, 1:1000), rabbit anti-Cleaved Caspase-3 (Cell Signaling Technology, 1:200), rabbit anti-Brn2 (Santa Cruz, 1:50), rabbit anti-CDP (Santa Cruz, 1:200), and rat anti-BrdU (Abcam, 1:1000). The secondary antibodies used here are Alexa Fluor 555 donkey anti-rabbit IgG (Invitrogen, 1:300), Alexa Fluor 488 donkey anti-rabbit IgG (Invitrogen, 1:300), Alexa Fluor 488 goat anti-rat IgG (Jackson ImmunoResearch, 1:300), Alexa Fluor 555 goat anti-rat IgG (Proteintech Group, 1:300). Nuclei were counterstained with 4, 6-diamidino-2-phenylindole (DAPI; Cayman Chemical).

Immunostaining images were collected using confocal laser microscopes (LSM 700 and LSM710, Carl Zeiss) or a light microscope (CTR 5000; Leica). The images were then analyzed with a Zeiss Aim Image Browser software or an Image-pro Plus software.

### BrdU labeling

BrdU was obtained from Sigma-Aldrich and dissolved in high pressure sterilized physiological saline solution at a concentration of 10 mg/ml. For the cell division experiment, the pregnant mice at E14.5 were injected intraperitoneally with BrdU at a concentration of 200 mg/kg for 2 h, during which time the progenitors in the S phase were labeled. For cell cycle analysis, the pregnant mice were intraperitoneally injected at E13.5 with BrdU at 50 mg/kg for 24 h, a time period required for cells to complete the entire cell cycle. Following injections, embryos were processed for immunohistochemistry as described above, except that the sections were treated with 2 M hydrochloric acid for 15 min at 45 °C for efficient BrdU staining.

### Cell count and statistical analysis

For embryonic studies, greater than 2 independent litters and 4 pairs of brains were used for immunostaining experiments. At least 3 sections of the medial cortex from each brain were analyzed for cell counts. For each section, an area of 300–400 μm wide with a length spanning either VZ/SVZ or the entire middle regions of the E14.5 telencephalon was used.

For postnatal brain analysis, greater than 3 littermates of each genotype were used for the experiment. And at least 3 discontinuous coronal sections from somatosensory cortex were counted. For the migration study, BrdU+ cells were measured in vertical strips with a 400 μm width and a length across the six-layered cortex and the strips were equally divided into ten bins throughout the six layers. The length of the cortex was identified by layer specific markers and the DAPI staining.

Student’s *t* test was used to evaluate the data statistically, and when P < 0.05, the differences were considered significant. And all data were presented as means ± standard errors of the means (SEM).

## Abbreviations

CNS: Central nervous system
BrdU: Bromodeoxyuridine
RGCs: Radial glia cells
IPCs: Intermediate progenitor cells
VZ: Ventricular zone
SVZ: Subventricular zone
CP: Cortical plate
IZ: Intermediate zone
APs: Apical progenitors
BPs: Basal progenitors
PAK1: P21-activated kinase 1
CDK5: Cyclin-dependent kinase 5
CDP: CUX1 (cut-like homeobox 1)
Brn2: Pou3f2 (POU domain, class 3, transcription factor 2)
Ctip2: Bcl11b (B cell leukemia/lymphoma 11 B)
Tbr1: T-box, brain 1
Pax6: Paired box 6
